# Evaluation of potential COVID-19 recurrence in patients with late repeat positive SARS-CoV-2 testing

**DOI:** 10.1371/journal.pone.0251214

**Published:** 2021-05-04

**Authors:** Ithan D. Peltan, Sarah J. Beesley, Brandon J. Webb, Bert K. Lopansri, Will Sinclair, Jason R. Jacobs, Samuel M. Brown

**Affiliations:** 1 Division of Pulmonary and Critical Care Medicine, Department of Medicine, Intermountain Healthcare, Salt Lake City, UT, United States of America; 2 Division of Pulmonary and Critical Care Medicine, Department of Internal Medicine, University of Utah School of Medicine, Salt Lake City, UT, United States of America; 3 Division of Infectious Disease and Clinical Epidemiology, Intermountain Healthcare, Salt Lake City, UT, United States of America; 4 Intermountain Laboratory Services, Department of Pathology, Intermountain Healthcare, Salt Lake City, UT, United States of America; University of Maryland School of Medicine, UNITED STATES

## Abstract

**Background:**

SARS-CoV-2 reinfection and reactivation has mostly been described in case reports. We therefore investigated the epidemiology of recurrent COVID-19 SARS-CoV-2.

**Methods:**

Among patients testing positive for SARS-CoV-2 between March 11 and July 31, 2020 within an integrated healthcare system, we identified patients with a recurrent positive SARS-CoV-2 reverse transcription polymerase chain reaction (RT-PCR) assay ≥60 days after an initial positive test. To assign an overall likelihood of COVID-19 recurrence, we combined quantitative data from initial and recurrent positive RT-PCR cycle thresholds—a value inversely correlated with viral RNA burden— with a clinical recurrence likelihood assigned based on independent, standardized case review by two physicians. “Probable” or “possible” recurrence by clinical assessment was confirmed as the final recurrence likelihood only if a cycle threshold value obtained ≥60 days after initial testing was lower than its preceding cycle threshold or if the patient had an interval negative RT-PCR.

**Results:**

Among 23,176 patients testing positive for SARS-CoV-2, 1,301 (5.6%) had at least one additional SARS-CoV-2 RT-PCRs assay ≥60 days later. Of 122 testing positive, 114 had sufficient data for evaluation. The median interval to the recurrent positive RT-PCR was 85.5 (IQR 74–107) days. After combining clinical and RT-PCR cycle threshold data, four patients (3.5%) met criteria for probable COVID-19 recurrence. All four exhibited symptoms at recurrence and three required a higher level of medical care compared to their initial diagnosis. After including six additional patients (5.3%) with possible recurrence, recurrence incidence was 4.3 (95% CI 2.1–7.9) cases per 10,000 COVID-19 patients.

**Conclusions:**

Only 0.04% of all COVID-19 patients in our health system experienced probable or possible recurrence; 90% of repeat positive SARS-CoV-2 RT-PCRs were not consistent with true recurrence. Our pragmatic approach combining clinical and quantitative RT-PCR data could aid assessment of COVID-19 reinfection or reactivation by clinicians and public health personnel.

## Introduction

The risk of recurrent COVID-19 resulting from SARS-CoV-2 reinfection or reactivation is currently unknown. Persistent detection of viral RNA for at least 30–60 days after patients’ initial positive assay is well described [1, 2], despite the fact that shedding of replication-competent virus appears to end within approximately 10–20 days of symptom onset [3–6]. The import of a positive SARS-CoV-2 assay beyond the 60–90 day mark, however, is less clear [7], especially given the symptomatic overlap between SARS-CoV-2 and other viral and bacterial infections. To date, SARS-CoV-2 reinfection and reactivation have mostly been described in case reports [8, 9] on the basis of confirmatory viral genotyping. However, this approach is often impractical for large scale analyses and for clinical application during the care of individual patients.

Understanding the epidemiology of COVID-19 recurrence is an urgent priority. To address this gap, we investigated patients in an integrated healthcare system who had a recurrent positive SARS-CoV-2 test ≥60 days after an initial positive test.

## Methods

### Study design, setting, and population

We performed a retrospective cohort study enrolling patients with positive SARS-CoV-2 reverse transcription polymerase chain reaction (RT-PCR) testing processed between March 11 and July 31, 2020 at laboratories operated by Intermountain Healthcare, an integrated healthcare system serving 1.5 million patients annually in Utah and southeastern Idaho that offered SARS-CoV-2 testing at 16 community drive-up testing sites, 32 urgent care clinics, and 23 hospitals/emergency departments during the study period. Analysis focused on patients with a recurrent positive SARS-CoV-2 RT-PCR ≥60 days after their initial positive test. We obtained demographic and clinical information and repeat SARS-CoV-2 testing results through October 27, 2020 via the health system’s electronic data warehouse, supplemented by manual review of the electronic medical record. Though developed prior to release of the Centers for Disease Control and Prevention common investigation protocol for suspected SARS-CoV-2 investigation [10], our data collection methods were largely consistent with this protocol.

The Intermountain Healthcare Institutional Review Board determined review of medical records for this study was exempt from review with waiver of informed consent.

### Clinical assessment of acute infection

Among patients with a positive SARS-CoV-2 RT-PCR performed ≥60 days after the initial positive assay, two physician reviewers independently adjudicated the clinical likelihood of acute COVID-19 at the time of both their initial positive test and their recurrent positive test (Table 1). For the initial positive test, adapting CDC case definition [11], we classified the likelihood of true positive infection as definite, probable, or possible. For the recurrent positive SARS-CoV-2 RT-PCR, adjudication into four categories of acute COVID-19 likelihood (probable, possible, unlikely, or very unlikely) was based on the presence of a clinical syndrome consistent with COVID-19, available evidence for interval clinical recovery, laboratory evidence for cleared infection, and documented new exposure. History of exposure to an individual known to have SARS-CoV-2 was documented as part of each testing order and was also obtained from clinical documentation when available. Patients were considered diagnosed with COVID-19 by their treating clinicians only if the diagnosis was explicit. A non-specific (e.g. “viral bronchitis”) or possible COVID-19 diagnosis was not considered a formal diagnosis. Physician reviewers could downgrade either the initial infection or recurrent COVID-19 likelihood based on factors including the presence/absence of alternative diagnoses or evidence that case had been reviewed at the time of treatment by a clinical expert (e.g. an infectious disease physician) who explicitly determined that patient did not have acute COVID-19. Reviewers were blinded to RT-PCR cycle thresholds (Ct).

**Table 1:** SARS-CoV-2 infection likelihood clinical adjudication criteria.

Adjudication topic	Clinical adjudication criteria
Adjudication of infection likelihood at time of initial positive SARS-CoV-2 RT-PCR	• *Definite*: clinical documentation c/w COVID-19 OR multiple symptoms (including 1 or more of cough, fever, SOB, anosmia/dysgeusia) • *Probable*: ≥1 symptom OR no documented symptoms AND either exposure OR high-risk situation (e.g. residence in a long-term care facility) • *Possible*: no documented symptoms AND no documented exposure AND no documented high-risk situation
Adjudication of acute COVID-19 at time of recurrent positive SARS-CoV-2 RT-PCR	• *Probable acute infection (ANY ONE)*: ○ Multiple symptoms AND either documented interval resolution, interval negative test, OR positive test interval >89 days after initial positive test ○ At least one specific symptom (cough, fever, SOB, anosmia) AND either documented interval resolution, interval negative test, OR positive test interval >89 days after initial positive test • *Possible acute infection (ANY ONE)*: ○ Multiple symptoms not meeting above criteria ○ At least one specific symptom (cough, fever, SOB, anosmia) ○ At least one non-specific symptom AND documented interval resolution, interval negative test, OR positive test interval >89 days after initial positive test ○ New exposure AND test interval >89 days • *Unlikely acute infection (ANY ONE)*: ○ Single symptom not meeting above criteria ○ New exposure OR positive interval >89 days OR interval negative test but no documented symptoms • *Very unlikely acute infection*: No documented symptoms AND no documented exposure AND positive test interval < 90 days

There was good interrater agreement regarding the likelihood of acute SARS-CoV-2 infection at both initial and recurrent positive testing (weighted kappa 0.77 [95% CI 0.64–0.88] and 0.72 [95% CI 0.63–0.80], respectively). Disagreement was resolved by consensus.

### Cycle threshold data

Ct values were obtained from SARS-CoV-2 RT-PCR processed via our healthcare system’s clinical laboratories. Several different testing platforms were used over the course of the study eligibility period (S1 Table). For RT-PCR assays reporting >1 Ct value per assay, we averaged the reported Ct values for use in evaluation of Ct trajectory.

### COVID-19 recurrence determination

**Fig 1:**
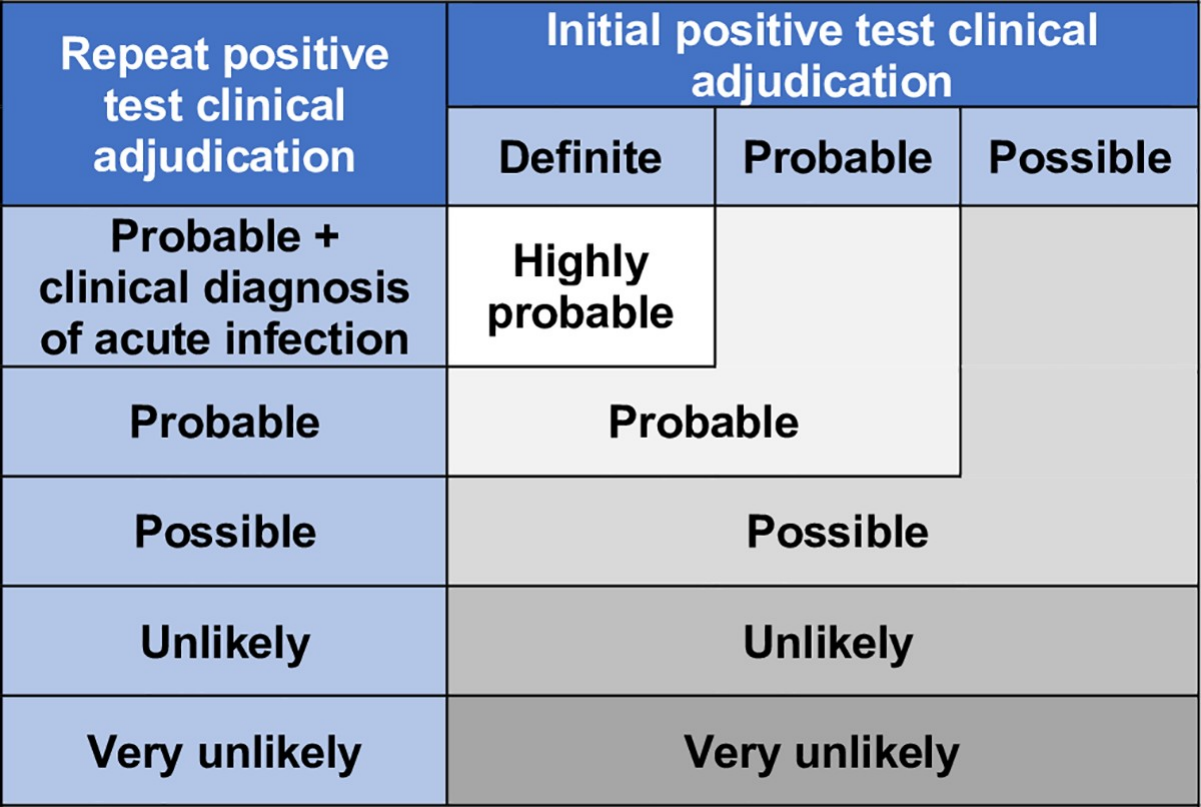
Algorithm for determining clinical likelihood of COVID-19 recurrence.

To evaluate the likelihood that each subject’s recurrent positive SARS-CoV-2 RT-PCR represented recurrent COVID-19, we first determined the *clinical* likelihood using a simple, conservative algorithm combining (1) the likelihood of true positive initial infection; (2) infection at repeat positive testing; and (3) diagnosis of COVID-19 at recurrent testing by patients’ treating clinicians (Fig 1). Categories were simplified for analysis into probable, possible, and unlikely.

**Fig 2:**
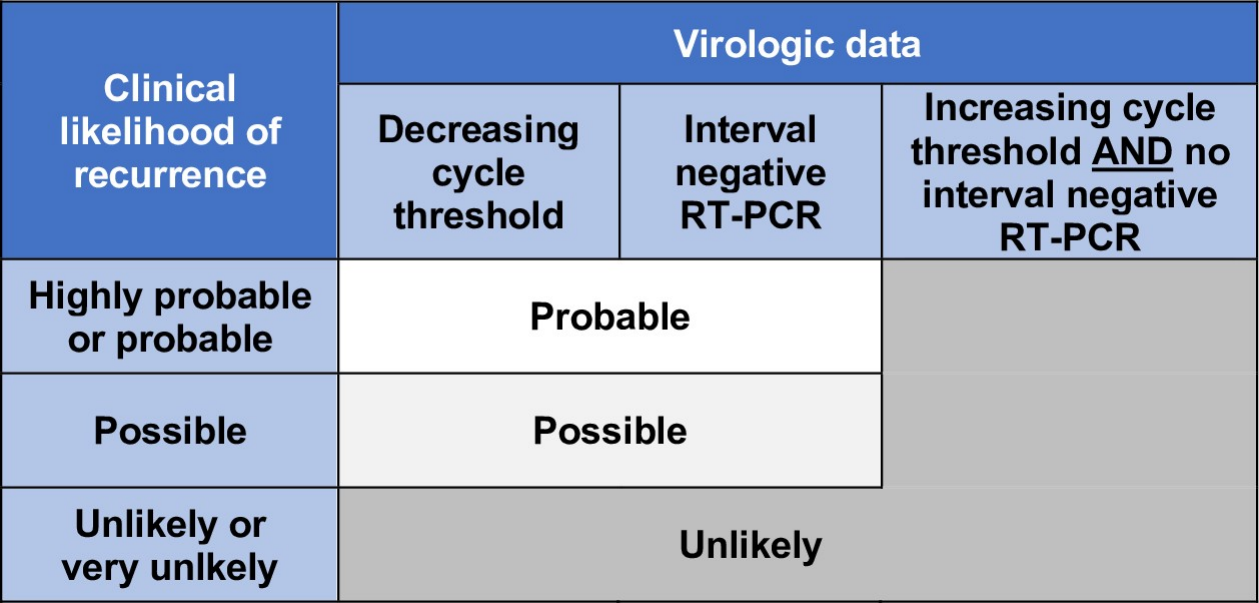
Algorithm for determining overall likelihood of COVID-19 recurrence.

The *overall* likelihood of recurrent COVID-19 was determined by prespecified criteria conservatively integrating the clinical recurrence assessment with Ct values, which correlate inversely with viral RNA burden (Fig 2). “Probable” or “possible” recurrence by clinical assessment was confirmed as the final recurrence likelihood only if a Ct value obtained ≥60 days after initial testing was lower than its preceding Ct or if the patient had an interval negative RT-PCR. We classified all other patients as “unlikely” recurrence. Interval negative tests—which by definition have higher Ct values than positive tests—were included to harness this qualitative indicator of Ct trajectory and avoid undercounting recurrence events. This strategy is also consistent with clinical practice and recent CDC analyses [12].

We performed three sensitivity analyses. First, to evaluate potential inter-assay variability, RT-PCR Ct trajectory were reevaluated after normalizing observed Ct values to the respective assays’ maximum Ct value. In the second sensitivity analysis, based on prior studies suggesting replication-competent virus is usually not present when RT-PCR Ct values are greater than 25–30 [5, 13], we added two additional criteria to the overall recurrence likelihood algorithm: (1) any patients whose lowest Ct value after 60 days was >30 was reclassified as “unlikely recurrence,” regardless of other factors; (2) patients with “unlikely” recurrence by clinical criteria were reclassified as “possible” recurrence if their lowest Ct value after 60 days was <25 *and* they had a decreasing Ct trajectory and/or an interval negative SARS-CoV-2 RT-PCR. Finally, in *post hoc* analyses, we (1) restricted the analysis to patients whose initial and recurrent testing employed the same testing platform and (2) applied an alternate COVID-19 recurrence definition adapted from Abu-Raddad et al. [14], classifying a recurrent positive RT-PCR with Ct <30 as “strong” evidence of recurrence and a recurrent positive RT PCR with Ct >30 associated with exposure, symptoms, or decreasing Ct trajectory as “good” evidence of recurrence.

### Data analysis

Data are reported as N (%) or median (interquartile range). We did not perform statistical hypothesis testing for this epidemiologic study. Overall recurrence incidence was calculated by dividing all patients with positive SARS-CoV-2 PCR by the number with an overall recurrence likelihood of “probable” or “possible.”

## Results

**Fig 3:**
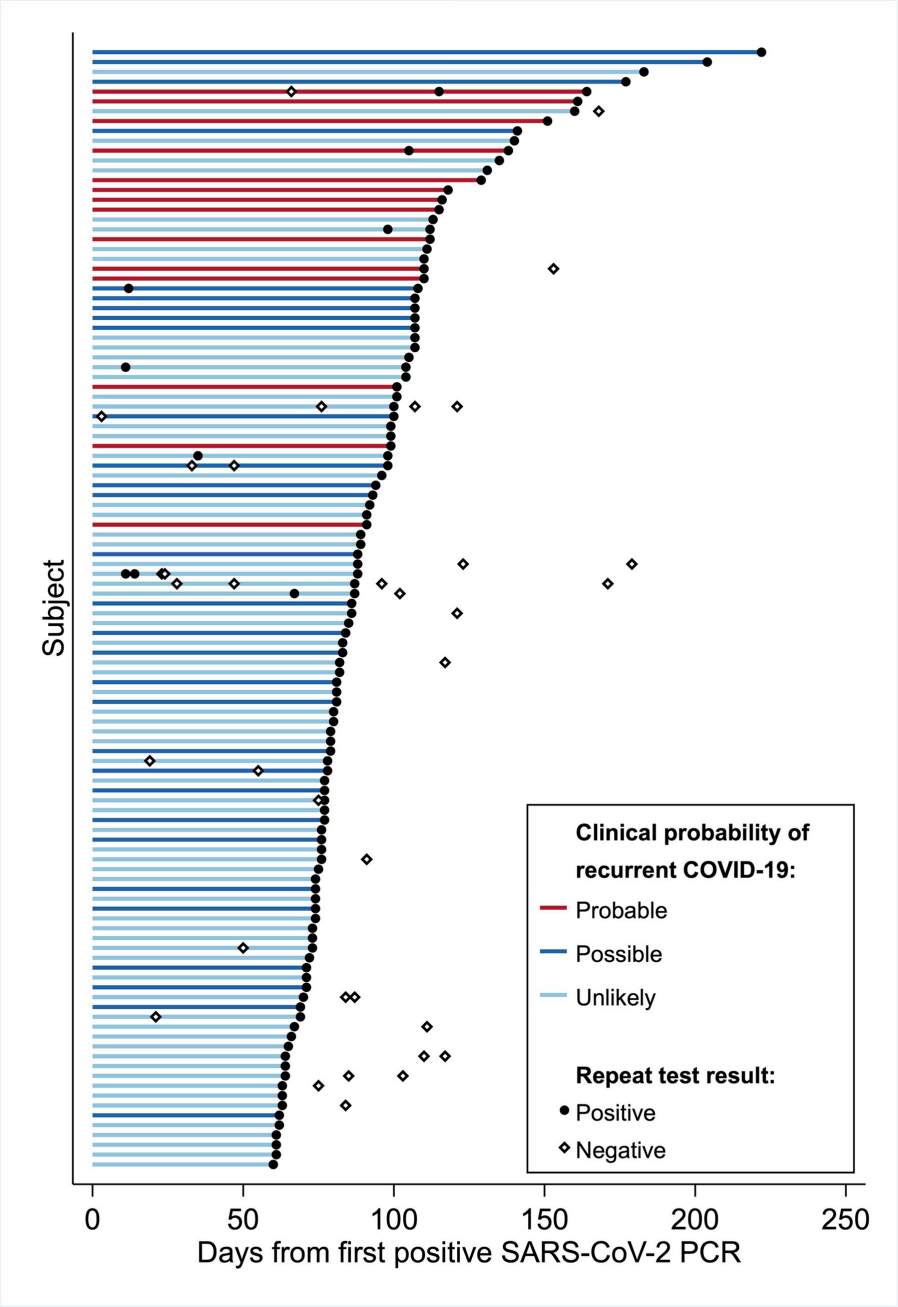
Interval from initial to recurrent positive SARS-CoV-2 RT-PCR. Horizontal lines depict the interval from the initial positive SARS-CoV-2 RT-PCR for each patient (N = 114) to their latest repeat positive test occurring at least 60 days subsequently as well as each subject’s clinical probability of COVID-19 recurrence. Individual RT-PCR tests occurring after subject’s initial positive test are shown as closed circles (positive result) and open diamonds (negative result).

Of 23,176 patients with a positive SARS-CoV-2 RT-PCR, 1,301 patients (5.6%) had one or more SARS-CoV-2 RT-PCRs collected ≥60 days later. 122 of the 1301 patients (9.4%) had a positive test, of whom 114 had initial and recurrent positive RT-PCR Ct values and sufficient clinical information for evaluation and were included in the present analysis (Table 2). Median interval to the recurrent positive test was 85.5 (74–107) days. No patients were immunocompromised, and six patients were younger than 18 years. The interval from the initial to recurrent positive test was ≥90 days for 49 patients (Fig 3). Patients in this group appeared more likely to have interval symptom recovery (95%) than patients whose recurrent positive assay occurred 60–89 days after their initial positive test (66%). Overall, recurrent COVID-19 was probable by purely clinical criteria in 14 patients (12.3%), possible in 30 (26.3%), and unlikely in 70 (61.4%, Table 3).

**Table 2:** Patient demographic and clinical characteristics.

	Overall (N = 114)		Positive SARS-CoV-2 test interval			
			60–89 days (N = 65)		≥90 days (N = 49)	
Age (years)	40	(26–56)	41	(27–59)	33	(26–55)
Female sex	64	(56.1%)	34	(52.3%)	30	(61.2%)
Race/ethnicity						
Hispanic or Latino	33	(28.9%)	20	(30.8%)	13	(26.5%)
Non-Hispanic Black	4	(3.5%)	0	(0%)	4	(8.2%)
Non-Hispanic White	68	(59.7%)	39	(60.0%)	29	(59.2%)
Other	9	(7.9%)	6	(9.2%)	3	(6.1%)
Charlson Comorbidity Index	0	(0–1)	0	(0–2)	0	(0–1)
Clinical COVID-19 disease at initial positive test						
Definite	89	(78.1%)	51	(78.5%)	38	(77.6%)
Probable	17	(14.9%)	10	(15.4%)	7	(14.3%)
Possible	8	(7.0%)	4	(6.2%)	4	(8.2%)
Care intensity associated with initial positive test						
Inpatient	14	(12.3%)	11	(16.9%)	3	(6.1%)
Outpatient (in-person or telemedicine)	20	(17.5%)	11	(16.9%)	9	(18.4%)
Test only	80	(70.2%)	43	(66.2%)	37	(75.5%)
SARS-CoV-2 RT-PCR cycle thresholds						
Initial positive	15.4	(11.0–20.6)	14.1	(11.4–17.5)	16.6	(11.0–21.9)
First recurrent positive after 60 or 90 days	32.5	(30.6–35.8)	32.6	(31.1–35.7)	32.3	(30.2–35.9)
Days to recurrent positive SARS-CoV-2 test	85.5	(74–107)	76	(69–81)	107	(100–118)
Negative interval SARS-CoV-2 test	11	(9.7%)	7	(10.8%)	4	(8.2%)
Interval symptom recovery^a^	76/95	(80.0%)	38/55	(69.1%)	38/40	(95.0%)
Disease indicator at repeat testing						
Symptoms and new exposure	19	(16.7%)	12	(18.5%)	7	(14.3%)
Symptoms only	34	(29.8%)	18	(27.7%)	16	(32.7%)
Exposure only	14	(12.3%)	7	(10.8%)	7	(14.3%)
None	47	(41.2%)	28	(43.1%)	19	(38.8%)
Care intensity associated with repeat positive test						
Inpatient	9	(7.9%)	5	(7.7%)	4	(8.1%)
Outpatient (in-person or telemedicine)	15	(13.2%)	6	(9.2%)	9	(18.4%)
Test only	90	(78.9%)	54	(83.1%)	36	(73.5%)
Clinical probability of COVID-19 recurrence						
Probable	14	(12.3%)	0	(0%)	14	(28.6%)
Possible	30	(26.3%)	17	(26.2%)	13	(26.5%)
Unlikely	70	(61.4%)	48	(73.8%)	22	(44.9%)

Values displayed as median (IQR) or N (%).

^a^ Restricted to patients with symptoms recorded at time of initial positive assay.

**Table 3:** Subject characteristics by clinical likelihood of recurrence.

	Clinical probability of recurrent COVID-19					
	Probable (N = 14)		Possible (N = 30)		Unlikely (N = 70)	
Age (years)	49	(26–57)	25	(20–38)	46	(30–61)
Female	9	(64.3%)	19	(63.3%)	36	(51.4%)
Race/ethnicity						
Hispanic or Latino	7	(50.0%)	13	(43.3%)	13	(18.6%)
Non-Hispanic Black	3	(21.4%)	0	(0%)	1	(1.4%)
Non-Hispanic White	2	(14.3%)	15	(50.0%)	54	*72.9%)
Other	2	(14.3%)	2	(6.7%)	5	(7.1%)
Charlson Comorbidity Index	0	(0–1)	0	(0–0)	1	(0–2)
Time to first recurrent positive SARS-CoV-2 test (days)	113.5	(105–118)	85	(77–107)	79.5	(71–98)
Documented symptoms at repeat testing	14	(100%)	24	(80.0%)	15	(21.4%)
Documented exposure at repeat testing	4	(28.6%)	15	(50.0%)	14	(20.0%)
SARS-CoV-2 RT-PCR cycle thresholds						
Initial positive	16.8	(10.8–22.7)	15.6	(11.4–21.9)	15.0	(10.8–19.1)
First recurrent positive on or after 60 days	42.7	(29.0–33.5)	31.8	(28.9–34.1)	33.7	(31.7–38.2)
Any cycle threshold <30 on or after 60 days	6	(42.9%)	10	(33.3%)	10	(14.3%)
Decreasing cycle threshold on repeat testing	4	(28.6%)	3	(10.0%)	2	(2.9%)
Interval negative SARS-CoV-2 RT-PCR	1	(7.1%)	3	(10.0%)	7	(10.0%)
Clinical diagnosis of SARS-CoV-2 reinfection	2	(14.3%)	0	(0%)	0	(0%)

Values displayed as median (IQR) or N (%).

**Fig 4:**
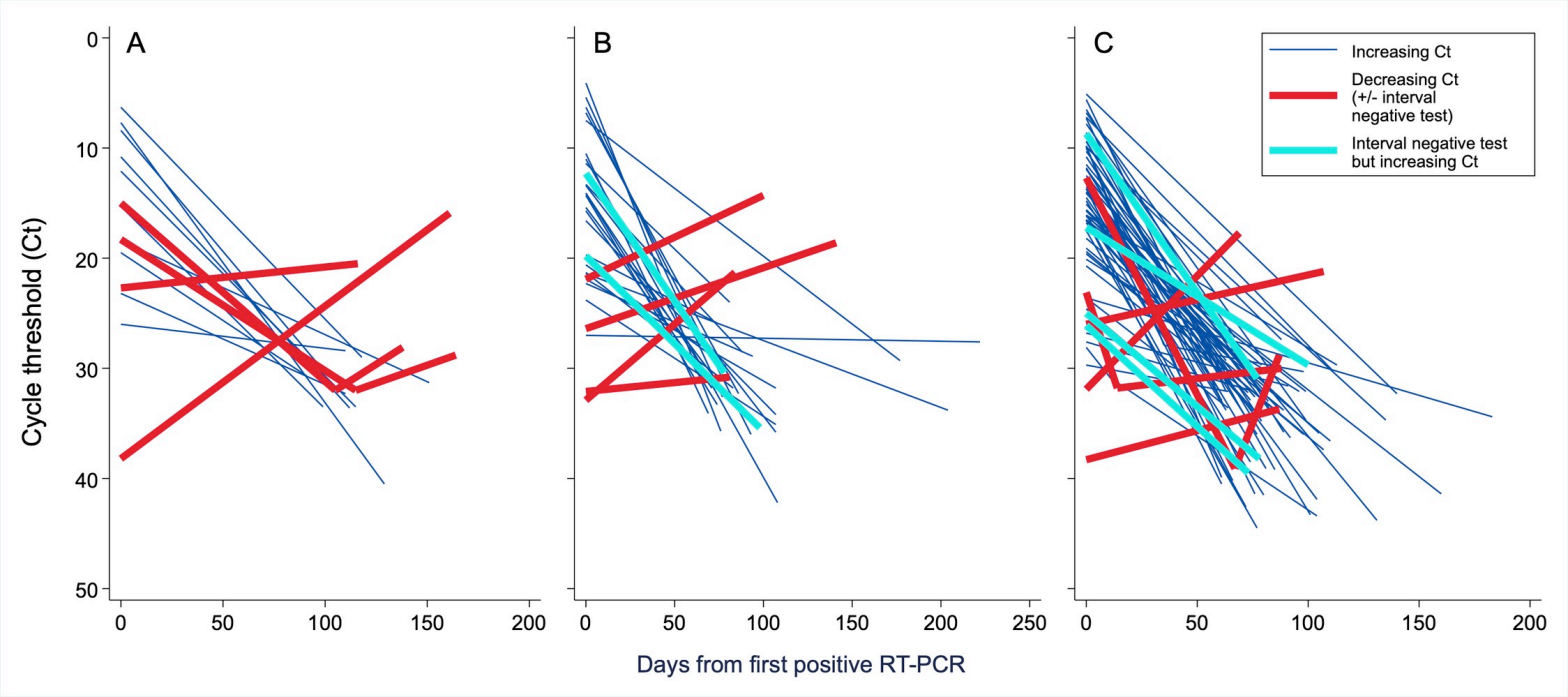
SARS-CoV-2 RT-PCR cycle threshold trajectory by clinical recurrence likelihood. Cycle threshold trajectory for each patient (N = 114) with a recurrent positive SARS-CoV-2 RT-PCR ≥60 days after their initial positive test, stratified by (A) probable (N = 14), (B) possible (N = 30), or (C) unlikely (N = 70) clinical likelihood of COVID-19 recurrence. Abbreviations: Ct, cycle threshold; Neg, negative; RT-PCR, reverse transcription polymerase chain reaction.

Ct values on repeat positive SARS-CoV-2 RT-PCR increased in most patients (Fig 4). Ten patients had negative interval RT-PCRs, eight had a subsequent Ct decrease, and one patient had both (Table 4). Documented COVID-19 exposure was more common among these patients (58%) compared to patients with increasing Ct values at repeat testing and no interval negative assay (23%). In the sensitivity analysis using Ct values normalized to the assay’s cycle threshold maximum, only one additional patient—whose clinical recurrence probability was classified as “unlikely”—had a decreasing Ct trajectory.

**Table 4:** Subject demographic characteristics by cycle threshold trajectory.

	Ct trajectory on repeat SARS-CoV-2 testing			
	Decreasing Ct or interval negative test (N = 19)		Increasing Ct (N = 95)	
Age (years)	40	(25–57)	40	(26–51)
Female	9	(47.4%)	55	(57.9%)
Race/ethnicity				
Hispanic or Latino	6	(31.6%)	27	(28.4%)
Non-Hispanic Black	2	(10.5%)	2	(2.1%)
Non-Hispanic White	10	(52.6%)	58	(61.1%)
Other	1	(5.3%)	8	(8.4%)
Charlson Comorbidity Index	0	(0–1)	0	(0–2)
Time to first recurrent positive SARS-CoV-2 test (days)	88	(78–107)	83	(74–107)
Documented symptoms at repeat testing	10	(52.6%)	43	(45.3%)
Documented exposure at repeat testing	11	(57.9%)	22	(23.2%)
Any cycle threshold <30 on or after 60 days	11	(57.9%)	15	(15.8%)
Clinical probability of COVID-19 recurrence				
Probable	4	(21.1%)	10	(10.5%)
Possible	6	(31.6%)	24	(25.2%)
Unlikely	9	(47.4%)	61	(64.2%)

Values displayed as median (IQR) or N (%).

Abbreviations: Ct, cycle threshold.

Four patients (3.5%) met criteria for a final determination of probable COVID-19 recurrence. All four patients had a Ct value <30 as well as symptoms at recurrence, three required a higher intensity of clinical care than at initial diagnosis, and two received inpatient treatment with an explicit diagnosis of recurrent COVID-19 by treating clinicians (Table 5). Six patients (5.3%) met criteria for possible recurrence, of whom three had Ct value <30 on their recurrent positive test, five had new or recurrent symptoms at recurrence and two received higher-intensity clinical care. The incidence of probable or possible COVID-19 recurrence was therefore 4.3 (95% CI 2.1–7.9) cases per 10,000 COVID-19 patients. Nine patients who were asymptomatic at recurrent positive testing and classified as unlikely recurrence had a decreasing Ct value or interval negative test. All nine were asymptomatic at the time of their recurrent positive testing.

**Table 5:** Clinical trajectory with interval negative SARS-CoV-2 RT-PCR or decreasing cycle threshold value on repeat RT-PCR.

	Symptoms or COVID-19 exposure			Highest associated clinical treatment intensity		RT-PCR data		
	Initial positive RT-PCR	Recurrent positive RT-PCR	Interval symptom recovery	Initial positive RT-PCR	Recurrent positive RT-PCR	Days to recurrent positive RT-PCR	Interval negative tests	Lowest Ct value after 60 days
Probable recurrence								
Patient A	Sx + Exp	Sx only	Yes	Testing only	Outpatient clinic	115	1	28.8
Patient B	Exp only	Sx only	N/A	Testing only	Inpatient ward	105	0	28.1
Patient C	Sx only	Sx + Exp	Yes	Testing only	ICU	116	0	20.5
Patient D	Sx only	Sx + Exp	Yes	Outpatient clinic	Outpatient clinic	161	0	15.9
Possible recurrence								
Patient E	Exp only	Sx + Exp	N/A	Testing only	Testing only	100	1	14.3
Patient F	Sx only	Exp only	Yes	Outpatient clinic	Testing only	141	0	18.6
Patient G	Sx + Exp	Sx + Exp	Yes	Testing only	Testing only	98	2	35.4
Patient H	Sx + Exp	Sx + Exp	Yes	Testing only	Testing only	84	0	21.3
Patient I	Sx only	Sx + Exp	Yes	Testing only	ICU	78	1	30.4
Patient J	Exp only	Sx only	N/A	Testing only	Outpatient clinic	81	0	30.8
Unlikely recurrence but interim negative or decreasing Ct value								
Patient K	Sx + Exp	Neither	Yes	Testing only	ICU	67	0	28.8
Patient L	Neither	Exp only	N/A	Testing only	Testing only	69	1	17.7
Patient M	Sx only	Neither	Yes	Testing only	Testing only	78	1	38.2
Patient N	Sx only	Neither	No	ICU	Telemedicine	73	1	39.5
Patient O	Sx only	Exp only	Unknown	ICU	Testing only	88	1	30.0
Patient P	Sx only	Exp only	Yes	Testing only	Testing only	87	2	33.7
Patient Q	Sx + Exp	Neither	Yes	Outpatient clinic	Testing only	100	1	29.7
Patient R	Sx + Exp	Neither	Yes	Outpatient clinic	Testing only	107	0	21.2
Patient S	Sx + Exp	Neither	Unknown	Testing only	Testing only	77	1	30.9

Values displayed as median (IQR) or N (%).

Abbreviations: Ct, cycle threshold; Exp, exposure; ICU, intensive care unit; N/A, not applicable; Sx, symptoms

In the sensitivity analysis incorporating Ct value-based thresholds into the recurrence likelihood assessment algorithm, three of the six patients classified as “possible” recurrence in the primary analysis were reclassified as unlikely recurrence because all Ct values from recurrent positive RT-PCRs were >30. Simultaneously, two patients classified as “unlikely” recurrence were reclassified as possible recurrence because at least one RT-PCR Ct value was <25 at least 60 days after their initial positive test in combination with a decreasing Ct trajectory and/or at least one interval negative test. After these reclassifications, the number of patients classified as possible recurrence decreased from six to five. Added to the four patients with probable recurrence (whose likelihood classification was not altered by the algorithm modifications), the sensitivity analysis therefore yielded an incidence of probable or possible COVID-19 recurrence of 3.8 (95% CI 1.8–7.4) cases per 10,000 COVID-19 patients. When restricted to the 42 patients whose initial and recurrent positive assay occurred on the same testing platform yielded, the rate of probable (2.4%) and possible recurrence (7.1%) were similar to the overall cohort.

More substantial differences in the estimated incidence were observed when using the COVID-19 recurrence definition adapted from Abu Raddad *et al*. By these criteria, 26 of our 114 patients with recurrent positive SARS-CoV-2 RT-PCRs had “strong” evidence of recurrence and 48 of 114 had “good” evidence of recurrence, yielding a COVID-19 recurrence incidence of 31.9 (95% CI 25.1–40.1) per 10,000 COVID-19 patients.

## Discussion

Though infectious SARS-CoV-2 shedding usually ends 10–20 days from symptom onset, RT-PCRs assays may remain positive for 8 weeks or longer [3, 15, 16]. Given COVID-19’s symptomatic overlap with other syndromes, this phenomenon poses major challenges for assessment of possible SARS-CoV-2 reinfection both clinically and epidemiologically. In this study, over 5% of COVID-19 patients had repeat SARS-CoV-2 testing 60 days or more after their initial positive test, of whom nearly 10% had a positive test result. However, we identified only 4 probable and 6 possible COVID-19 recurrences—0.04% of patients with an initial positive SARS-CoV-2 RT-PCR—after combining clinical data (recurrent clinical syndrome consistent with COVID-19, testing interval, and re-exposure) and patients’ viral load trajectory. Although reinfection seems more likely in these immunocompetent patients, reactivation is also possible.

We found higher COVID-19 recurrence rates compared to a recent study measuring subsequent SARS-CoV-2 RT-PCR positivity among seropositive healthcare workers [17]. We suspect this study undercounted COVID-19 recurrences among patients who failed to generate a measurable SARS-CoV-2 antibody response. By contrast, the recurrence rate observed in our health system was similar to a recent population-based study from Qatar by Abu-Raddad *et al*. [14]. This similarity, however, occurred despite important differences in recurrence case definition. The analysis by Abu-Raddad *et al*. prioritized the recurrent positive RT-PCR’s Ct as a single indicator for identifying COVID-19 recurrence, backed up by a fairly liberal symptom-based case definition. In particular, dependance on surrogate markers to identify COVID-19 exposure and symptoms may have underestimated the prevalence of these indicators in the Qatar-based cohort. Applied to our health system’s patients, this approach increased the estimated COVID-19 recurrence incidence by nearly an order of magnitude. This discrepancy simultaneously highlights the limitations of recurrence definitions based solely on Ct values (given Ct variation across the COVID-19 disease course [2] and between assays, laboratories, and specimen collection methods [18–20]), and, on the other hand, the importance of comprehensive clinical evaluation when incorporating symptoms and exposure as criteria for COVID-19 recurrence.

A possible limitation of our approach, depending on application, is that it is designed to undercount asymptomatic reinfection. Our algorithm emphasized clinical and epidemiological criteria and largely precluding diagnosis of asymptomatic infection even when the molecular evidence was seemingly strong. In fact, several patients classified as unlikely recurrence given the absence of a concurrent clinical syndrome exhibited a down-trending Ct trajectory and a very low Ct value at recurrence. Whether these patients had asymptomatic recurrence, reinfection, or merely persistent shedding of viral RNA is unknown. Future analyses applying both traditional and molecular epidemiology analyses will be needed to better define the incidence of asymptomatic COVID-19 recurrence and associated transmission rates. Nevertheless, a strategy similar to the one used in our sensitivity analysis—in which recurrence probability was downgraded in patients with very high Ct values at recurrence and upgraded in patients with very low Ct values—may be useful for public health and epidemiologic purposes.

Our study is limited by a lack of viral genotype data, the gold standard method for diagnosing SARS-CoV-2 reinfection. Compared to genetic analysis [8], our assessment algorithm may miscategorize as COVID-19 recurrence syndromes due to other pathogens superimposed on prolonged asymptomatic shedding. Because routine viral genotyping is often not currently feasible in real time to guide clinical care and remains challenging for large-scale epidemiologic analyses, however, our pragmatic approach could aid further urgent evaluation of COVID-19 reinfection or reactivation by clinicians and public health personnel.

This study has several additional limitations. In addition to undercounting asymptomatic reinfection, our analysis will miss serial testing events in other healthcare systems (the frequency of which is unknown). We will also undercount recurrence in patients whose specimens were of poor quality and some patients without an interval negative tests who have low viral loads at repeat testing despite true recurrence. The CDC common investigation protocol for COVID-19 recurrence—released shortly after our study protocol was developed—suggests investigating patients with a positive test ≥45 days after their first positive rather than ≥60 days as was done here. However, the CDC protocol simultaneously suggests prioritizing investigation of potential recurrence occurring ≥90 days from the initial positive test, and the relatively low frequency of probable or possible recurrence in the 60–89 day versus ≥90 day window suggests few probable or possible recurrences would have been identified in the 45–59 day window. Finally, while the sensitivity analyses that used normalized Ct values and restricted evaluation to patients tested serially on the same platform are reassuring, between-platform variation in gene targets, Ct ranges, and other assay parameters may have influenced the apparent Ct trajectories among patients whose recurrent test took place on a different platform than the original test [18–20].

## Conclusions

Recurrent COVID-19 affected only 0.04% of patients with an initial positive SARS-CoV-2 assay in our health system. Neither clinical criteria nor Ct assessment alone appeared sufficient to accurately categorize likelihood of COVID-19 recurrence. Combining clinical quantitative RT-PCR data with clinical criteria provides may serve as a practical framework for categorizing potential recurrence cases as the number of recovered COVID-19 patients rises, immunity wanes, and the probability reinfection events increases.

## Supporting information

S1 Table SARS-CoV-2 RT-PCR assays. (PDF)
