# Supplementary material for: Evaluation of potential COVID-19 recurrence in patients with late repeat positive SARS-CoV-2 testing
Source: PLoS One. 2021 May 4;16(5):e0251214. doi: 10.1371/journal.pone.0251214 (PMC8096096; doi:10.1371/journal.pone.0251214)
Supplement: S1 Table — (PDF) [file pone.0251214.s001.pdf]

S1 Table

ONLINE SUPPLEMENT

EVALUATION OF POTENTIAL COVID-19 RECURRENCE IN PATIENTS WITH LATE REPEAT POSITIVE SARS-COV-2 TESTING

Ithan D. Peltan, MD, MSc, Sarah J. Beesley, MD, MS, Brandon J. Webb, MD,
Bert K. Lopansri, MD, Will Sinclair, BS, Jason R. Jacobs, PhD, Samuel M. Brown, MD, MS

S1 Table. SARS-CoV-2 RT-PCR assays.

Assay manufacturer	Genes targeted[a]	Ct values reported per test	Maximum Ct value for assay	Assays performed[b]	Positive[b]	Ct value available[b]
Centers for Disease Control [1,2]	N (3 targets)[c]	3	40	3	3	3
Quidel [3]	ORF	1	30	51	46	44
Cepheid [4]	N, E	2	45	32	32	31
Thermo Fisher Scientific [5]	ORF, N, S	3	40	195	164	164
Hologic [6]	ORF (2 targets)	1	40	7	7	7
Biofire [7]	ORF (3 targets)	N/A[d]	N/A[d]	3	3	0
Roche [8]	ORF, E	2	40	4	2	2

Abbreviations: Ct, cycle threshold; ORF, open reading frame

[a] SARS-CoV-2 genes targeted by utilized assays were the those encoding the structural nucleocapsid (N), envelope (E), and spike (S) proteins and the open reading frame (ORF) polyprotein.[9]

[b] Includes RT-PCR assays (positive and negative) performed for patients with ≥1 recurrent positive RT-PCR assay ≥60 days after their initial positive assay.

[c] At the time this assay was used, CDC assay employed three targets. The CDC RT-PCR has subsequently been reduced to two targets.

[d] Cycle threshold values are not reported for this assay.

1. Holshue ML, DeBolt C, Lindquist S, Lofy KH, Wiesman J, Bruce H, et al. First Case of 2019 Novel Coronavirus in the United States. New Engl J Med. 2020;382: 929–936. doi:10.1056/NEJMoa2001191
2. CDC 2019 Novel Coronavirus (nCoV) Real-Time RT-PCR Diagnostic Panel — Instructions for Use. In: U.S. Food Drug Administration [Internet]. 12 Jul 2020 [cited 18 Nov 2020]. Available: https://www.fda.gov/media/134922/download
3. Lyra® SARS-CoV-2 Assay Instructions for Use. In: Quidel [Internet]. 21 May 2020 [cited 18 Nov 2020]. Available: https://www.quidel.com/sites/default/files/product/documents/PIM120002EN00_10_20_Lyra_SARS-CoV-2_Assay_Pkg_Insert_EUA.pdf
4. Xpert Xpress SARS-CoV-2 Assay Instructions for Use. In: Cepheid [Internet]. Sep 2020 [cited 18 Nov 2020]. Available: https://www.cepheid.com/Package%20Insert%20Files/Xpress-SARS-CoV-2/Xpert%20Xpress%20SARS-CoV-2%20Assay%20ENGLISH%20Package%20Insert%20302-3750-Xpress%20Rev.%20E.pdf
5. TaqPath COVID-19 Combo Kit: Instructions for Use. In: ThermoFisher Scientific [Internet]. 15 Mar 2020 [cited 18 Nov 2020]. Available: https://assets.thermofisher.com/TFS-Assets/LSG/manuals/MAN0019181_TaqPath_COVID-19_IFU_EUA.pdf
6. SARS-CoV-2 Assay (Panther Fusion® System). In: Hologic [Internet]. 3 Apr 2006 [cited 18 Nov 2020] pp. 1–34. Available: https://www.hologic.com/sites/default/files/2020-09/AW-21159-001_004_01.pdf
7. BioFire COVID-19 Test Instructions for Use. In: BioFire Defense [Internet]. 5 Oct 2020 [cited 18 Nov 2020]. Available: https://www.biofiredefense.com/wp-content/uploads/2020/04/BioFire-COVID-19-Instructions-for-Use-DFA2-PRT-0080-03.pdf
8. Poljak M, Korva M, Knap Gašper N, Fujs Komloš K, Sagadin M, Uršič T, et al. Clinical Evaluation of the cobas SARS-CoV-2 Test and a Diagnostic Platform Switch during 48 Hours in the Midst of the COVID-19 Pandemic. J Clin Microbiol. 2020;58. doi:10.1128/JCM.00599-20
9. Tang Y-W, Schmitz JE, Persing DH, Stratton CW. Laboratory diagnosis of COVID-19: current issues and challenges. J Clin Microbiol. 2020;58. doi:10.1128/JCM.00512-20
